# Root Zone Cooling and Exogenous Spermidine Root-Pretreatment Promoting *Lactuca sativa* L. Growth and Photosynthesis in the High-temperature Season

**DOI:** 10.3389/fpls.2016.00368

**Published:** 2016-03-23

**Authors:** Jin Sun, Na Lu, Hongjia Xu, Toru Maruo, Shirong Guo

**Affiliations:** ^1^College of Horticulture, Nanjing Agricultural UniversityNanjing, China; ^2^Center for Environment, Health and Field Sciences, Chiba UniversityChiba, Japan

**Keywords:** high temperature, root zone cooling, spermidine, photosynthesis, chlorophyll fluorescence, *Lactuca sativa* L.

## Abstract

Root zone high-temperature stress is a major factor limiting hydroponic plant growth during the high-temperature season. The effects of root zone cooling (RZC; at 25°C) and exogenous spermidine (Spd) root-pretreatment (SRP, 0.1 mM) on growth, leaf photosynthetic traits, and chlorophyll fluorescence characteristics of hydroponic *Lactuca sativa* L. grown in a high-temperature season (average temperature > 30°C) were examined. Both treatments significantly promoted plant growth and photosynthesis in the high-temperature season, but the mechanisms of photosynthesis improvement in the hydroponic grown lettuce plants were different between the RZC and SRP treatments. The former improved plant photosynthesis by increasing stoma conductance (*G*_s_) to enhance CO_2_ supply, thus promoting photosynthetic electron transport activity and phosphorylation, which improved the level of the photochemical efficiency of photosystem II (PSII), rather than enhancing CO_2_ assimilation efficiency. The latter improved plant photosynthesis by enhancing CO_2_ assimilation efficiency, rather than stomatal regulation. Combination of RZC and SRP significantly improved *P*_N_ of lettuce plants in a high-temperature season by both improvement of *G*_s_ to enhance CO_2_ supply and enhancement of CO_2_ assimilation. The enhancement of photosynthetic efficiency in both treatments was independent of altering light-harvesting or excessive energy dissipation.

## Introduction

Temperature is one of the most important environmental factors affecting plant growth and development (Levitt, 1980). The responses of plant growth and photosynthesis to different air temperatures have been intensively studied (Kratsch and Wise, 2000; Wise et al., 2004; Way and Yamori, 2014). However, studies on a plant’s response to root zone temperature remain limited. Root zone temperature is important for plant growth as it greatly affects various growth processes, and is more critical than air temperature in the control of plant growth (Xu and Huang, 2000). In hydroponic systems, the nutrient solution temperature often differs from ambient air temperature (Zheng et al., 1993; Nagasuga et al., 2011). The use of root temperature management can strongly influence plant growth in various species, such as holly (*Ilex chinensis Sims*; Ruter and Ingram, 1992), cotton (*Gossypium* spp; McMichael and Burke, 1994), and bean (*Phaseolus acutifolius* and *Proteus vulgaris*) (Udomprasert et al., 1993). Some studies reported that plant photosynthesis could be enhanced by reducing the root zone temperature. For example, photosynthetic rate of ‘Rotundifolia’ holly (*I. crenata* Thunb.) plants grown with root zones at 38 or 42°C was lower than that of at 30 or 34°C (Ruter and Ingram, 1992). Photosynthetic rate in *Lactuca sativa* plants was higher in root-zone temperature of 20°C condition than that of hot ambient temperature (He et al., 2009). Therefore, in addition to reducing greenhouse air temperature, an appropriate root temperature management methodology may effectively regulate plant photosynthesis, growth and yield with relatively low investment and easier management. Alternatively, increasing plant heat tolerance using plant growth regulators is another option for growing plants in high-temperature seasons.

Polyamines (PAs) are low-molecular-weight aliphatic amine bases with strong biological activity. They are important regulators of plant growth, stress response, and disease resistance (Hussain et al., 2011). In higher plants, the most common PAs are spermidine (Spd), spermine (Spm), and putrescine (Put). PAs are involved in higher plant growth, morphogenesis, and anti-aging regulation, and are also closely related to the ability of a plant to resist adversity stress (Moschou and Roubelakis-Angelakis, 2014). Recently, much attention has been paid to the relationship between PAs and plant responses to abiotic stress. Polyamines can alleviate salt stress on *Arabidopsis* growth (Ortega-Amaro et al., 2012), improve salt resistance in pea (*Pisum sativum* L.) plants (Pottosin et al., 2014), and increase the heat tolerance of wheat (*Triticum aestivum* L.; Asthir et al., 2012), rice (*Oryza sativa*; Mohammad et al., 2014), and cucumber (*Cucumis sativus* L.) plants (Tian et al., 2012). Exogenous Spd pretreatment improves cold resistance of cucumber plants (He et al., 2002b), and improves tolerance to cadmium stress in bulrushes (*Typha latifolia* L.; Zacchini et al., 2003). Additionally, a significant improvement of heat tolerance is observed in genetically modified tomatoes (*Solanum lycopersicum*) with overexpressed PAs, confirming PAs are involved in plant response to heat stress (Cheng et al., 2009). Attentions have been paid to the effects of application of exogenous PAs on plant photosynthesis under various stresses conditions. It has been demonstrated that exogenously applied PAs can rapidly enter the intact chloroplast (He et al., 2002a) and play a role in protecting the photosynthetic apparatus from adverse effects of environmental stresses (Navakoudis et al., 2003). Exogenous PAs improved the photosynthetic capacity of salt-stressed cucumber plants by increasing the level of the photochemical efficiency of photosystem II (PSII; Zhang et al., 2009). In green alga (*Scenedesmus obliquus*) cultivation, exogenously added Put was used to adjust the increase in the functional size of the antenna and the reduction in the density of active PSII reaction centers, so that to confer some kind of tolerance to the photosynthetic apparatus to against enhanced NaCl-salinity and permit cell growth even in NaCl concentrations that under natural conditions would be toxic (Demetriou et al., 2007). Investigations on restoration of the maximum photochemical efficiency (*F*_v_/*F*_m_) to low salt stressed thylakoid by adding Put, Spd and Spm showed that Spd are the most efficient one in *F*_v_/*F*_m_ restoration (Ioannidis and Kotzabasis, 2007). When *Physcia semipinnata* was exposed to UV-A radiation, it was also found that exogenously Spd applied samples had higher chlorophyll *a* content and PSII activity than Spm and Put applied samples (Unal et al., 2008).

Lettuce (*L. sativa* L.) is one of the most commonly cultivated crops used in hydroponics systems. In the summer, high temperatures in the greenhouse are the most significant limiting factor for lettuce production. During the midday period, in the hot summers of the Tokyo region of Japan, the root zone temperature of hydroponic systems often exceeds 30°C (e.g., the root zone temperature can often reach 35°C when the air temperature is 38°C); this strongly suppresses the lettuce growth process and reduces production. Lettuce production is significantly reduced at 30–35°C root zone temperatures compared with when grown at 25°C (Li et al., 2015), with the maximum dry mass of lettuce obtained under 24°C/24°C (air/root zone temperature) conditions (Thompson et al., 1998). Therefore, there is a possibility to improve the growth of lettuce plants in the high-temperature season by decreasing the root zone temperature or enhancing plant heat tolerance using exogenous plants growth regulators.

Photosynthesis is essential for plant growth and development, and an improvement of leaf photosynthesis would lead to enhancement of crop yield (Ainsworth and Long, 2005; Ainsworth, 2008; Yamori et al., 2016). Chlorophyll fluorescence analysis, as one of methods of studies in photosynthesis, is one of the most powerful and widespread techniques, which can serve as a sensitive indicator of thylakoid membranes damage and functional changes of photosynthetic apparatus under high temperature stress (Murkowski, 2001). However, information regarding the effects of root zone cooling (RZC) and exogenous PAs/Spd root-pretreatment (SRP) on the photosynthetic characteristics of plants in hydroponic system or the possible alleviation mechanisms of RZC and SRP on the negative effects of high temperature season is limiting in literature currently. The objective of this study is to investigate the effects of RZC and SRP (root-soaking) on hydroponic lettuce growth, photosynthesis, and chlorophyll fluorescence characteristics during the high-temperature season. The photosynthetic physiological mechanisms of RZC and SRP on plant growth are discussed.

## Materials and Methods

### Plants

Experiments were carried out in a glass greenhouse (36 m length, 18 m width, 3.9 m height; North–South oriented) at the Center for Environment, Health and Field Sciences in Chiba University (N 35°53′, E 139°56′) Japan, from July 15 to August 16, 2015. Lettuce (*L. sativa* L., cv. Romaine) seeds were sown in sponge blocks (W 2.3 cm × D 2.3 cm × H 2.7 cm) on June 24, 2015 and transferred into a germination room (20°C) for 2 days. Following germination, seedlings were transferred into a growth chamber (Nae Terrace, Mitsubishi Plastics Agri Dream Co., Ltd.) under a 12-h photoperiod with 350 ± 10 μmol m^-2^ s^-1^ light intensity, 22/18°C day/night temperature, and 1000 ppm CO_2_ concentration for 19 days. Next, morphologically uniform lettuce seedlings were transferred into the greenhouse, and planted on foam boards floating on containers (volume 90 L, 16 plants per container). Each container was equipped with a water temperature control system and air pump to supply fresh air to the nutrient solution [Otsuka formula (**Table 1**)], electrical conductivity (EC): 2.0 ± 0.2 dS m^-1^, pH: 6.0 ± 1.0. During the cultivation period, the average air temperature was 36.6°C/28.3°C (day/night), average relative humidity was 63.5%, and average daily photosynthetic photo flux density (PPFD) was 561 μmol m^-2^ s^-1^ inside greenhouse.

**Table 1:** Otsuka formula.

Composition	Content (%)	Composition	Content (%)
*N*	21.0	Fe	0.18
P_2_O_5_	8.0	Cu	0.002
K_2_O	27	Zn	0.006
MgO	4.0	Mo	0.002
MnO	0.10		
B_2_O_3_	0.10		
CaO	23.0		

### Experimental Design

The RZC and SRP experiments were conducted when the 14th leaf had fully expanded, 7 days after planting. There were four test set treatments: (1) Plants cultivated at a 30°C root zone temperature without SRP, as a control (30°C); (2) Plants cultivated at a 30°C root zone temperature, with SRP (30°C + Spd); (3) Plants cultivated at a 25°C root zone temperature, without SRP (25°C); and (4) A combination of 25°C root zone temperature and SRP (25°C + Spd). The containers were arranged in a 2-way factorial complete block design with three blocks, comprising a total of 12 containers with 192 seedlings in the four treatments (48 seedlings per treatment). The experiment was conducted in the center area of the greenhouse where the environment conditions are relatively uniform. Root zone temperature control was realized using a heater (Ic Auto Neo Type 180, Nisso, Japan) or a cooler (Compact Handy Cooler 202TCN, As One, Japan) in the nutrient solution of each container. According to empirical data for the Tokyo region, the root zone temperature often reaches 30°C in the summer season, and a previous study found 25°C to be the optimal temperature for growth of ‘cv. Romaine’ lettuce (Li et al., 2015). Thus 30°C was selected as the control, and 25°C as the RZC treatment. For SRP, plant roots were soaked in 0.1 mM Spd (Sigma, St Louis, MO, USA; 99.9% purity) solution for 15 min, then the liquid was drained on the root surface until there was no dripping, after which the plant roots were planted back to the foam boards. To investigate the direct relationship between Spd and lettuce photosynthesis, six sub-treatments at six different Spd concentrations (0, 0.01, 0.05, 0.1, 0.15, and 0.2 mM) were conducted during experimental period. The method was as described above, except for the changed Spd concentrations. The root zone temperature for sub-treatment plants was 30°C. Three plants were randomly selected from each container for gas-exchange parameters and chlorophyll fluorescence measurements at 9:00–11:00 a.m. on 19 days after planting (12 days after treatment), and eight plants were randomly selected from each container for plant growth measurements on 21 days after planting. The measurement of photosynthesis or chlorophyll fluorescence was carried out using intact plants, and parameters were measured on a fully expanded functional leaf at the same position of a plant for each treatment.

### Temperature in the Greenhouse, and Root Zone Temperature Measurement

The air temperature inside the greenhouse and root zone temperature under control conditions (30 and 25°C) were recorded using thermo recorders (TR–71wf, T&D, Japan). For the measurement of root zone temperatures, sensors were located 10 cm deep inside the nutrient solution. Temperature data were recorded at 10-min intervals during the experimental period, and daily temperatures were calculated using a 24-h average.

### Plant Growth Analyses and Chlorophyll Content Measurement

For determination of fresh weight, the plants were washed with distilled water and weighed after wiping the water off. The number of fully expanded leaves was recorded as leaf number per plant. Leaf area per plant was measured using a Li–3000 leaf area meter (Li–Cor, Lincoln, NE, USA). Leaf chlorophyll content was determined using a chlorophyll meter (SPAD–502, Minolta, Osaka, Japan). Samples were oven dried at 70°C until a constant weight was attained, and the dry weight subsequently recorded.

### Gas-Exchange Parameter Measurements

The measurement method of Gas-exchange parameters was the same as described previously (Yamori et al., 2011) using a portable photosynthesis system (Li–6400XT, Li-Cor Inc., Lincoln, NE, USA) between 09:00 and 11:00. Net photosynthetic rate (*P*_N_), stomatal conductance (*G*_s_), transpiration rate (*T*_r_), and intercellular CO_2_ concentration (*C*_i_) were measured. Light was provided from red and blue light-emitting diodes (6400–02B, Li-Cor Inc.). Photosynthetic photon flux density (PPFD) was measured at 800 μmol m^-2^ s^-1^, and the leaf temperature, CO_2_ concentration, and relative humidity (RH) were 28 ± 1°C, 400 ± 2 μmol mol^-1^, and 63 ± 2%, respectively.

### Light Response and CO_2_ Response Curve Measurements

Leaf temperature was set at 25°C, and PPFD settings were 1,600, 1,400, 1,200, 1,000, 800, 600, 400, 200, 150, 100, 50, 25, and 0 μmol photons m^-2^ s^-1^. The *P*_N_–PPFD curve was plotted using *P*_N_ data and the corresponding light intensity. When measuring the CO_2_ response curve, leaf temperature was set at 25°C, and CO_2_ concentrations were set to 1,200, 1,000, 800, 600, 400, 200, 150, 100, 50, 25, and 0 μmol mol^-1^, the light intensity was set at 800 μmol m^-2^ s^-1^. The *P*_N_–Ci curve was then plotted in accordance with the *P*_N_ data and corresponding CO_2_ concentration.

The *P*_N_–PPFD and *P*_N_–Ci curves were fitted with least-squares according to Bassman and Zwier (1991), to obtain light-saturated maximum photosynthetic rate (*P*_Nmax_), apparent quantum yield (AQY), CO_2_-saturated maximum photosynthetic rate (*A*_max_), and carboxylation efficiency (CE). *P*_Nmax_ was determined as the maximum net photosynthetic rate at saturation light intensity, AQY was determined as the initial slope of the *P*_N_–PPFD curves, *A*_max_ determined as the maximum net photosynthetic rate at a saturated CO_2_ concentration, and CE was determined as the initial slope of the *P*_N_–Ci curves.

### Chlorophyll Fluorescence Parameter Measurements

Chlorophyll fluorescence parameters were measured to evaluate the light absorption, transfer, dissipation, and distribution in the photosystem of lettuce plants treated by RZC or SRP after leaves adaption in light or dark to a stable state. Leaf chlorophyll fluorescences were measured simultaneously using a portable photosynthesis system (Li–6400XT, Li-Cor Inc.) with an integrated fluorescence fluorometer (Li 6400–40 leaf chamber fluorometer, Li-Cor Inc.) under ambient CO_2_ concentrations and 21% O_2_. Actinic light supplied by light-emitting diodes (90% red light, 630 nm; 10% blue light, 470 nm) was used to record the steady state chlorophyll fluorescence level (*F*_s_). The minimum chlorophyll fluorescence at the open PSII center (*F*_o_) and maximum chlorophyll fluorescence at the closed PSII center (*F*_m_) were measured after 30 min of dark adaptation. Measurement light (630 nm, 1 μmol m^-2^ s^-1^) was used to determine *F*_o_. An 800-ms saturating pulse (>6,000 μmol m^-2^ s^-1^) was applied to measure *F*_m_ in the dark or during actinic light illumination (F′_m_). The minimum (F′_o_) fluorescence of light-adapted leaves was determined in accordance with Kramer et al. (2004), that the actinic light was put out and then the minimal fluorescence level in the light-adapted state was determined by illuminating the leaf with a 3-s far-red light. The maximum quantum yield of the PSII primary photochemistry (*F*_v_/*F*_m_) was calculated as (*F*_m_–*F*_o_)/*F*_m_. The quantum yield of PSII electron transport [PhiPSII = (F′_m_ - F_s_)/F′_m_], the efficiency of excitation energy capture by open PSII reaction centers [*F′_v_/F′_m_ = (F′_m_ - F′_o_)/F′_m_*], photochemical quenching [*qP = (F′_m_ - F_s_)/(F′_m_ - F′_o_)*], and non-photochemical quenching [qN = (F_m_ - F′_m_)/(F_m_ - F′_o_)] were calculated from the measured parameters (Maxwell and Johnson, 2000). The quantum yield of the carboxylation rate (PhiCO_2_) was calculated as: PhiCO_2_ = (*P*_N_–*P*_Ndark_)/(I × αleaf; Thwe et al., 2014), where *P*_N_ is the assimilation rate, *P*_N__dark_ is the dark assimilation rate (μmol m^-2^ s^-1^), and α is the initial slope of the light curve at low PPFD.

### Chlorophyll Fluorescence Kinetics Curves and the Fluorescent–CO_2_ Response Curve Measurements

Chlorophyll fluorescence kinetics curves were measured to understand the changes of chlorophyll fluorescence parameters when the lettuce leaves were suddenly transferred from dark to light, and the fluorescent–CO_2_ response curves were also measured to clarify the effect of CO_2_ concentration on chlorophyll fluorescence parameters, which would help to understand the changes of PhiPSII, PhiCO_2_, and *q*P in lettuce leaves treated with RZC and SRP. Chlorophyll fluorescence measurements were recorded from the fluorescence light curve and the fluorescence CO_2_ response curve of light-adapted leaves. Leaf chlorophyll fluorescences were measured simultaneously using a portable photosynthesis system (Li–6400XT, Li-Cor Inc.), with an integrated fluorescence fluorometer (Li 6400–40 leaf chamber fluorometer, Li-Cor Inc.). The fully expanded functional leaf at the same position of each plant was wrapped in foil paper and transferred to a dark place for a 12-h dark adaptation. Operation of the chlorophyll fluorescence induction kinetic auto-measurement program set the *F*_o_ and *F*_m_ at 547 and 2465, respectively (these values were obtained when measuring chlorophyll fluorescence parameters); the dark respiration rate was set at 0.45, which was obtained from the light response curve measurement. Loop N times and time between flashes were set at 10 times and 3 min, respectively. Saturated light intensity was set to 1,000 μmol m^-2^ s^-1^. The PhiPSII and PhiCO_2_ data were recorded for plotting the chlorophyll fluorescence induction kinetic curve. For plotting the fluorescent–CO_2_ response curve, settings were as follows: modulation frequency: 0.25 KHz; duration: 0.8 s; average signal frequency: 1 KHz; flash irradiance: 6,000 μmol m^-2^ s^-1^; modulation frequency: 20 KHz; and average signal frequency: 50 KHz. The light-adapted leaf from each treatment used the prior settings [*F*_o_ (547), *F*_m_ (2465), and dark respiration rate (0.45)] obtained from the control condition. The CO_2_ concentrations were set at 0, 50, 100, 150, 200, 400, 600, 800, and 1,000 μmol mol^-1^. PhiPSII and PhiCO_2_ data under different CO_2_ concentrations were recorded for plotting the fluorescent–CO_2_ response curve.

### Statistical Analysis

All experiments were conducted using three biological replicates. Eight plants from each replicate were selected for plant growth analyses, and three plants from replicate were selected for determination of gas-exchange parameters and chlorophyll fluorescence. Data were represented as the mean ± standard errors (SE). All data were statistically analyzed with SAS software (SAS Institute, Cary, NC, USA) using the Duncan’s multiple range test at *P* < 0.05 level of significance.

## Results

### Temperature in the Greenhouse and Root Zone

**FIGURE 1:**
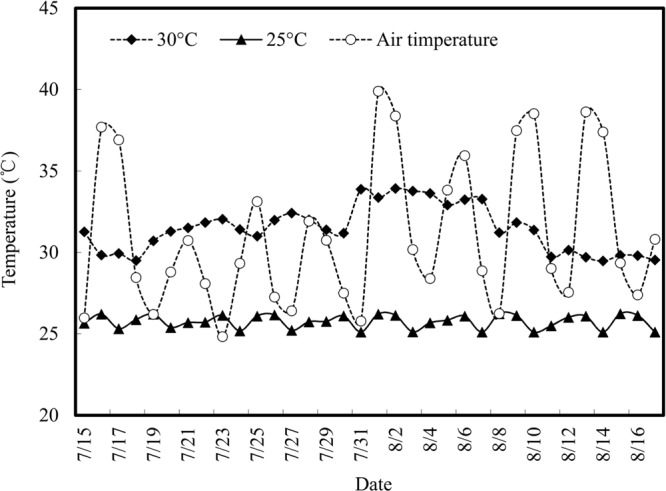
**Variation of air temperatures in the greenhouse and root zone temperature during the experimental period (15 July to 16 August 2015).** Root zone temperatures were measured for the 30°C treatment and 25°C treatment.

Air and root zone temperatures during the experimental period are shown in **Figure 1**. Diurnal air temperature varied intensely, with natural fluctuation. The highest air temperature reached was 38.6°C and the daily average temperature was 31.1°C. The root zone temperature showed less fluctuation, being relatively stable under temperature control treatments. The average root zone temperature of the control group (with a set point of 30°C) was 31.5°C, and the average temperature for RZC treatment was 25.8°C (with a set point of 25°C), which were within the expected experimental temperature range.

### Plant Growth and Chlorophyll Content

Root zone cooling and SRP exerted positive effects on plant growth. Compared with control group plants, RZC increased plant shoot fresh weight, root fresh weight, shoot dry weight, root dry weight, total plant fresh weight, and total plant dry weight by 8.9, 20.5, 7.8, 14.3, 9.7, and 8.5%, respectively; total leaf area and leaf number were unchanged (**Table 2**).

**Table 2:** Effects of root zone cooling (RZC) and exogenous Spd root-pretreatment on lettuce plant growth and chlorophyll content.

Treatment	Shoots fresh weight (g)	Roots fresh weight (g)	Shoots dry weight (g)	Roots dry weight (g)	Total fresh weight (g)	Total dry weight (g)	Leaf area (cm^2^)	Leaf number per plant	SPAD
30°C	108.45 ± 6.76c	8.33 ± 0.73c	4.11 ± 0.06d	0.54 ± 0.03c	116.78 ± 7.89c	4.65 ± 0.16c	2014.24 ± 23.94c	20.7 ± 0.85b	41.8 ± 1.77a
30°C + Spd	145.37 ± 4.25a	12.20 ± 0.42a	5.62 ± 0.43a	0.74 ± 0.07a	157.57 ± 4.67a	6.37 ± 0.50a	2258.68 ± 115.53b	22.3 ± 1.22a	44.3 ± 3.21a
25°C	118.06 ± 3.54b	10.04 ± 0.62b	4.43 ± 0.14c	0.61 ± 0.03b	128.10 ± 5.16b	5.05 ± 0.38b	2048.21 ± 35.04c	21.3 ± 1.04ab	42.5 ± 2.15a
25°C + Spd	141.87 ± 4.30a	12.51 ± 0.54a	5.00 ± 0.11b	0.70 ± 0.02a	154.38 ± 4.84a	5.69 ± 0.18a	2483.02 ± 73.22a	23.0 ± 2.11a	45.3 ± 2.09a

*Each value is the mean ± SE of three replicates. Different letters indicate significant differences at *P* < 0.05 according to Duncan’s multiple range test. 30°C: control, plants cultivated at a 30°C root zone temperature; 30°C + Spd: plants cultivated at the control root zone temperature with 0.1 mM Spd root-pretreatment; 25°C: plants cultivated at a 25°C root zone temperature; 25°C + Spd: combined 25°C root zone temperature and 0.1 mM Spd root-pretreatment. SPAD: leaf chlorophyll content determined using a SPAD chlorophyll meter.*

Under the control 30°C root zone temperature condition, SRP increased shoot fresh weight, root fresh weight, shoot dry weight, root dry weight, total plant fresh weight, total plant dry weight, total leaf area, and leaf number by 34.1, 46.4, 36.7, 38.5, 34.9, 36.9, 12.1, and 8.1%, respectively, compared with the control group plants (**Table 2**). Under 25°C root zone temperature conditions, SRP also significantly promoted plant growth. However, chlorophyll content (SPAD) was not affected by either RZC or SRP.

### Gas-Exchange Parameters

**FIGURE 2:**
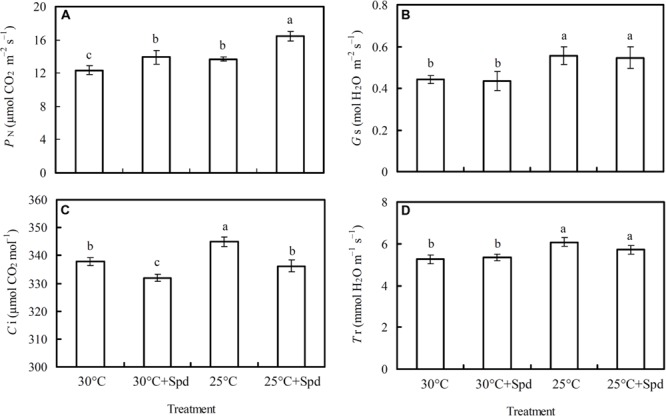
**Effects of root zone cooling (RZC) and exogenous spermidine (Spd) root-pretreatment on **(A)** net photosynthetic rate (*P*_N_); **(B)** stomatal conductance (*G*_s_); **(C)** intercellular CO_2_ concentration (*C*_i_); and **(D)** transpiration rate (*T*_r_) in leaves of hydroponic lettuce plants grown in a high-temperature season.** Parameters were measured on a fully expanded leaf at the same position for each treatment. Data represent mean ± SE (*n* = 3). Different letters indicate significant differences at *P* < 0.05 according to Duncan’s multiple range test. 30°C: control, plants cultivated at a 30°C root zone temperature; 30°C + Spd: plants cultivated at the control root zone temperature with 0.1 mM Spd root-pretreatment; 25°C: plants cultivated at a 25°C root zone temperature; 25°C + Spd: combined 25°C root zone temperature and 0.1 mM Spd root-pretreatment.

Root zone cooling significantly increased plant leaf net photosynthetic rate (*P*_N_; **Figure 2A**), stomatal conductance (*G*_s_; **Figure 2B**), intercellular CO_2_ concentration (*C*_i_; **Figure 2C**), and transpiration rate (*T*_r_; **Figure 2D**). SRP significantly increased *P*_N_ and decreased *C*_i_, whereas no significant effects on *G*_s_ and *T*_r_ were observed under both root zone temperature conditions (control and RZC).

### Light Response Curve and CO_2_ Response Curve

**FIGURE 3:**
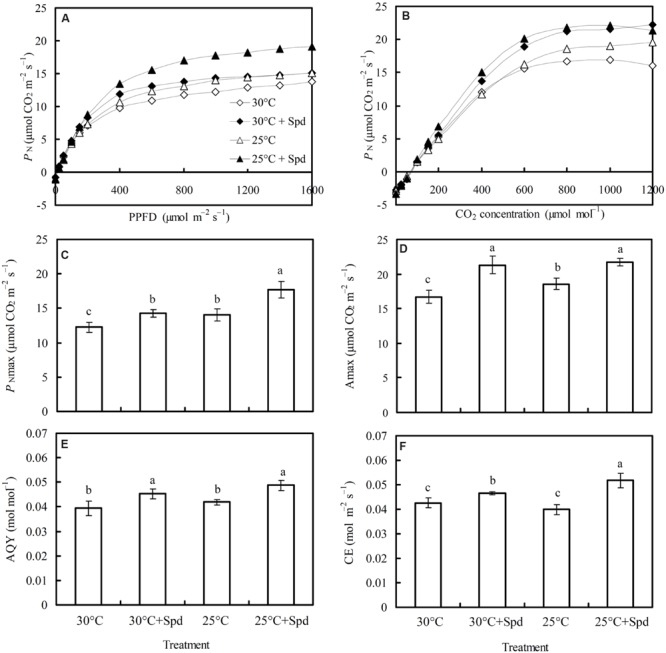
**Effects of RZC and exogenous spermidine (Spd) root-pretreatment on **(A)** light response curve (*P*_N_–PPFD); **(B)** CO_2_ response curve (*P*_N_–CO_2_); **(C)***P*_Nmax_; **(D)***A*_max_; **(E)** apparent quantum yield (AQY), and **(F)** carboxylic efficiency (CE) in the leaves of hydroponic lettuce plants grown in a high-temperature season.** Parameters were measured on a fully expanded leaf at the same position for each treatment. Data represent mean ± SE (*n* = 3). Different letters indicate significant differences at *P* < 0.05 according to Duncan’s multiple range test. 30°C: control, plants cultivated at a 30°C root zone temperature; 30°C + Spd: plants cultivated at the control root zone temperature with 0.1 mM Spd root-pretreatment; 25°C: plants cultivated at a 25°C root zone temperature; 25°C + Spd: combined 25°C root zone temperature and 0.1 mM Spd root-pretreatment.

*P*_N_ increased with the increase of PPFD, flattening after light intensity had reached 1,000 μmol m^-2^ s^-1^, indicating that the light saturation point for lettuce plant is approximately 1,000 μmol m^-2^ s^-1^ (**Figure 3A**). The *P*_N_ also increased CO_2_ concentration, flattening once the CO_2_ concentration reached 800 μmol mol^-1^, indicating that the CO_2_ saturation point for lettuce is approximately 800 μmol mol^-1^ (**Figure 3B**).

Root zone cooling significantly increased light-saturated maximum photosynthetic rate (*P*_Nmax_; **Figure 3C**) and CO_2_-saturated maximum photosynthetic rate (*A*_max_; **Figure 3D**), by 14.6 and 11.0%, respectively, whereas no significant effect was observed on apparent quantum yield (AQY; **Figure 3E**) or carboxylation efficiency (CE; **Figure 3F**). Under 30°C root zone temperature conditions (control), SRP significantly increased *P*_Nmax_, *A*_max_, AQY, and CE by 16.8, 27.3, 15.1, and 9.5%, respectively. Under 25°C root zone temperature conditions, SRP increased *P*_Nmax_ by 26.6%, *A*_max_ by 17.2%, AQY by 16.2%, and CE by 29.9%.

### Effects of Different Spd Concentrations on Plant Photosynthesis Traits

**FIGURE 4:**
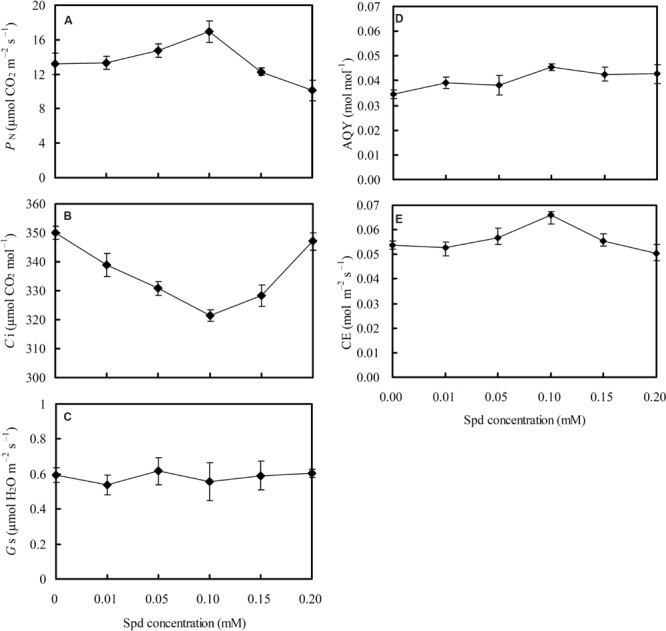
**Effects of different exogenous spermidine (Spd) concentrations on **(A)** net photosynthetic rate (*P*_N_); **(B)** intercellular CO_2_ concentration (*C*_i_); **(C)** stomatal conductance (*G*_s_); **(D)** apparent quantum yield (AQY); and **(E)** carboxylic efficiency (CE) in leaves of hydroponic lettuce plants grown in a high-temperature season.** Parameters were measured on a fully expanded leaf at the same position for each treatment. Data represent mean ± SE (*n* = 3).

Photosynthesis traits under different Spd concentrations of root-pretreatment were measured to analyze the direct relationship between Spd and lettuce CO_2_ assimilation efficiency (**Figure 4**). *P*_N_ initially increased and then decreased subsequently with the increase in Spd concentrations (**Figure 4A**), whereas *C*_i_ initially decreased and then increased subsequently with increased Spd concentrations (**Figure 4B**); *P*_N_ was negatively correlated with *C*_i_ (*r* = -0.6955^∗^, asterisk represents significant difference at 0.05). AQY (**Figure 4D**) and CE (**Figure 4E**) showed the same trend as seen for *P*_N_. Moreover, *P*_N_ showed significantly positive consistency with changes of CE (*r* = 0.8994^∗∗^, asterisks represent significant difference at 0.01), and *C*_i_ showed significantly negative consistency with changes of CE (*r* = -0.8242^∗^). However, *G*_s_ was not affected by Spd concentration (**Figure 4C**), and had no significant correlation with *P*_N_.

### Chlorophyll Fluorescence Parameters

**FIGURE 5:**
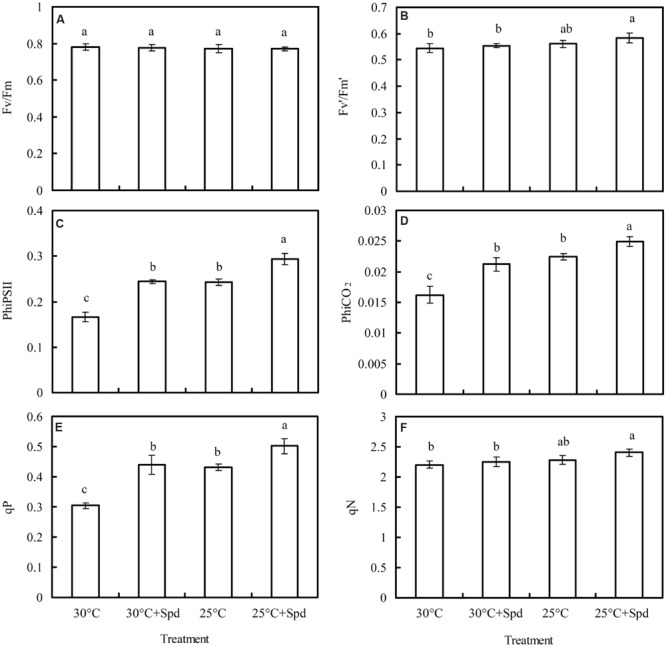
**Effects of RZC and exogenous spermidine (Spd) root-pretreatment on **(A)***F*_v_/*F*_m_; **(B)** F′_v_/F′_m_; **(C)** PhiPSII; **(D)** PhiCO_2_; **(E)***q*P; and **(F)***q*N in leaves of hydroponic lettuce plants grown in a high-temperature season.** Parameters are measured on a fully expanded leaf at the same position for each treatment. Data represent mean ± SE (*n* = 3). Different letters indicate significant differences at *P* < 0.05 according to Duncan’s multiple range test. 30°C: control, plants cultivated at a 30°C root zone temperature; 30°C + Spd: plants cultivated at the control root zone temperature with 0.1 mM Spd root-pretreatment; 25°C: plants cultivated at a 25°C root zone temperature; 25°C + Spd: combined 25°C root zone temperature and 0.1 mM Spd root-pretreatment.

Root zone cooling significantly increased quantum yield of PSII electron transport (PhiPSII; **Figure 5C**), quantum yield of the carboxylation rate (PhiCO_2_; **Figure 5D**), and photochemical quenching (*q*P; **Figure 5E**), but had no effect on maximum quantum yield of the PSII primary photochemistry (*F*_v_/*F*_m_; **Figure 5A**), efficiency of excitation energy capture by open PSII reaction centers (*F′_v_/F′_m_*; **Figure 5B**), or non-photochemical quenching (*q*N; **Figure 3F**). Under 30°C and RZC conditions, SRP significantly increased PhiPSII, PhiCO_2_, and *q*P, but had no effect on *F*_v_/*F*_m_, *F′_v_/F′_m_*, or *q*N.

### Chlorophyll Fluorescence Kinetics Curves and Fluorescent–CO_2_ Response Curve

**FIGURE 6:**
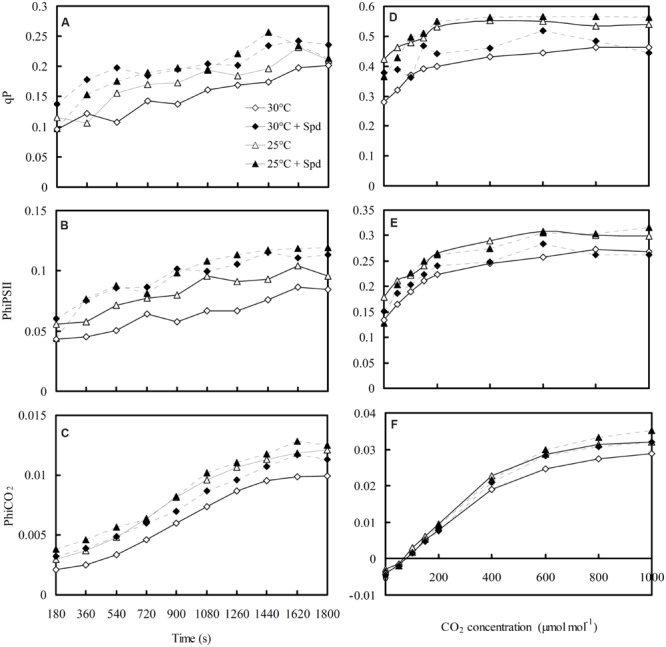
**Effects of RZC and exogenous spermidine (Spd) root-pretreatment on chlorophyll fluorescence kinetics curves **(A–C)** during a 30-min activated process from dark to light, and on fluorescent–CO_2_ response curves **(D–F)** of light-adapted leaves.** Parameters were measured on a fully expanded leaf at the same position for each treatment. Data represent mean ± SE (*n* = 3). 30°C: control, plants cultivated at a 30°C root zone temperature; 30°C + Spd: plants cultivated at the control root zone temperature with 0.1 mM Spd root-pretreatment; 25°C: plants cultivated at a 25°C root zone temperature; 25°C + Spd: combined 25°C root zone temperature and 0.1 mM Spd root-pretreatment.

Dynamic changes of *q*P (**Figure 6A**), PhiPSII (**Figure 6B**), and PhiCO_2_ (**Figure 6C**) of lettuce plants during 30 min of activated process from dark to light were measured and fluorescent–CO_2_ response curves plotted (**Figures 6D–F**). The *q*P, PhiPSII, and PhiCO_2_ initially increased, and began to flatten out with increased activation time and elevated CO_2_ concentrations. Both RZC and SRP increased *q*P, PhiPSII, and PhiCO_2_ of the lettuce leaf.

## Discussion

### Both RZC and SRP Improved Hydroponic Lettuce Growth during the High Temperature Season

Air and nutrient solution temperatures during the experimental period were both over 30°C (**Figure 1**), this is beyond the optimal temperature for hydroponic ‘cv. Romaine’ lettuce growth (Li et al., 2015), and thus limited plant production. In the present study, lettuce growth was remarkably improved by RZC (**Table 2**), proving the feasibility of cultivating hydroponic lettuce in a high-temperature season through cooling of the nutrient solution. Our results also demonstrated the growth of lettuce could be effectively promoted by application of 0.1 mM Spd (**Table 2**), thus providing a new way to improve hydroponic lettuce growth during the high-temperature season.

### RZC and SRP Improved *P*_N_ of Hydroponic Lettuce Plants in High Temperature Season via Different Mechanisms from Aspect of Gas-Exchange Parameters

Photosynthesis is extremely sensitive to supra-optimal temperatures, which can damage the first metabolic process (Paulsen, 1994). Many factors can cause decreased photosynthesis under biotic stress; possible reasons for this include the following: (1) damaged photosynthetic apparatus (Del Duca et al., 1994; Tiburcio et al., 1994; Borrell et al., 1995; He et al., 2002a; Demetriou et al., 2007); (2) degraded photosynthetic pigments (Aldesuquy et al., 2000; Subhan and Murthy, 2001; Chattopadhayay et al., 2002; Unal et al., 2008; Shu et al., 2011); (3) CO_2_ was prevented from entering into the mesophyll cell because of stoma closure (Liu et al., 2000; Shi et al., 2010); and (4) carbon assimilation was suppressed (Iqbal and Ashraf, 2005; Zhang et al., 2009; Shu et al., 2011, 2014). Our results showed that RZC improved *P*_N_ of hydroponic lettuce plants in high temperature season, similar results also found in several other species that photosynthesis were improved when reduced soil temperature (Martin et al., 1989; Udomprasert et al., 1993). However, the chlorophyll content in lettuce leaves was not affected by RZC (**Table 2**) when plants were exposed to a high-temperature season, indicating the integrity of the photosynthetic apparatus and light harvest efficiency were not affected. Therefore, the increased *P*_N_ by RZC probably due to CO_2_ supply being improved by stomatal conductance or/and enhancement of CO_2_ assimilation. According to the determination of stomatal and non-stomatal limitation described by Farquhar and Sharkey (1982), the *P*_N_ (**Figure 2A**), *G*_s_ (**Figure 2B**), *C*_i_ (**Figure 2C**), and *T*_r_ (**Figure 2D**) of lettuce plants in this study were increased by RZC, suggesting the increase of *P*_N_ was caused by improved stomatal conductance, which enabled sufficient CO_2_ for photosynthesis. This result agreed with Dodd et al. (2000), who found that when transfer pepper (*Capsicum annuum* L.) plants from root zone temperature of 25–40°C to 20°C condition, *G*_s_ was increased, and RZC likely improved root hydraulic conductivity independently on water viscosity, alternation in hydraulic conductivity have in turn increased shoot water potential, which is hypothesized to have directly enhanced the stomatal aperture. He et al. (2001) and Zhang et al. (2008) also suggested that changes in photosynthesis induced by root temperatures were mainly attributed to corresponding changes of *G*_s_.

However, the *P*_N_ of lettuce plants was also significantly increased by SRP when plants were exposed to both the control and RZC, but *C*_i_ was decreased, and *G*_s_ and *T*_r_ were not affected. These results suggest the increase of *P*_N_ in lettuce plants by SRP correlates to the improvement of CO_2_ assimilation, rather than to the CO_2_ supply being enhanced by stomatal conductance. Studies on the effects of PAs on plant photosynthesis in other species showed similar mechanisms: application of Put foliar spray on cucumber plants under salt stress (Zhang et al., 2009), Spd foliar spray on rice plants under drought stress (Farooq et al., 2009), and Spd foliar spray on corn plants under salt stress (Liu et al., 2006), all showed photosynthesis improvements correlated to carboxylic efficiency. However, in those studies, *G*_s_ was suppressed by PAs application, which helps to decrease transpiration and enhance water use efficiency of plants under adverse stress conditions (Yuan et al., 2012). In this study, *G*_s_ (**Figure 2B**) was not affected by SRP, probably because the Spd treatment was conducted on plant roots rather than leaves.

Therefore, the gas-exchange parameter data indicated the mechanisms of *P*_N_ improvement in lettuce plants between RZC and SRP were different, the former owing to the improvement of CO_2_ supply, while the latter ascribable to CO_2_ assimilation enhancement.

### Apparent Photosynthetic Parameters from the *P*_N_–PPFD and *P*_N_–C_i_ Curves Provided Further Evidence that Improvement Mechanisms of *P*_N_ were Different between RZC and SRP When Lettuce Plants were Exposed to High Temperature

The *P*_N_–PPFD (**Figure 3A**) and *P*_N_–*C*_i_ (**Figure 3B**) curves of hydroponic lettuce plants were determined to further understand differences in the mechanisms of *P*_N_ improvement between RZC and SRP. Apparent photosynthetic parameters, including *P*_Nmax_ (**Figure 3C**), *A*_max_ (**Figure 3D**), AQY (**Figure 3E**), and CE (**Figure 3F**), were calculated to understand the response of photosynthesis in lettuce plants to the above two treatments. *P*_Nmax_ represents the maximum net photosynthetic rate at saturation light intensity, while *A*_max_ represents the maximum net photosynthetic rate at a saturated CO_2_ concentration, and *A*_max_ correlates with the activities of photosynthetic electron transport and phosphorylation (Coste et al., 2005). AQY represents CO_2_ assimilation or O_2_ release when a plant absorbs one photon, and CE represents carboxylic efficiency, which positively correlates to Ribulose-1,5-bisphosphate carboxylase/oxygenase (Rubisco) activity (Reng et al., 2003). The results revealed the *P*_Nmax_ (**Figure 3C**) and *A*_max_ (**Figure 3D**) of lettuce plants were significantly increased by RZC, whereas AQY (**Figure 3E**) and CE (**Figure 3F**) were not affected, suggesting that *P*_N_ improvement of plants by RZC correlated with the promotion of photosynthetic electron transport activity and phosphorylation, rather than enhancement of CO_2_ assimilation efficiency. However, *P*_Nmax_, *A*_max_, AQY, and CE were all increased by SRP at both 30 and 25°C root zone temperatures, suggesting that *P*_N_ improvement by SRP not only correlated with promotion of photosynthetic electron transport activity and phosphorylation, but also correlated with enhancement of CO_2_ assimilation efficiency. Meanwhile, results of different Spd concentrations on *P*_N_ (**Figure 4A**), *C*_i_ (**Figure 4B**), *G*_s_ (**Figure 4C**), AQY (**Figure 4D**), and CE (**Figure 4E**) of lettuce plants further interpreted the mechanism of SRP on photosynthesis improvement. There was a significant negative correlation between *P*_N_ and *C*_i_, and a positive correlation between *P*_N_ and CE, whereas no correlation was observed between *P*_N_ and *G*_s_. These findings provide further evidence that improvement of *P*_N_ by SRP is due to enhancement of carboxylic efficiency when lettuce plants are exposed to the control and RZC, but is not due to stomatal regulation.

CO_2_ assimilation of plants is regulated by Rubisco activity and RuBP regeneration (Farquhar and Sharkey, 1982; Wise et al., 2004). Rubisco activity is described by CE (Reng et al., 2003), while RuBP regeneration depends on production of ATP and NADPH, which correlate with photosynthetic electron transport and phosphorylation (Coste et al., 2005). Therefore, *A*_max_ also reflects RuBP regeneration. In the present study, RZC increased *A*_max_ (**Figure 3D**) without affecting the CE (**Figure 3F**) of lettuce plants, suggesting that the improvement in photosynthesis correlated with increased RuBP regeneration, but not Rubisco activity. Loss of photosynthetic activity at high shoot and leaf temperatures has been related to decreased Rubisco activity (Ruter and Ingram, 1992), which is different from our deduction, probably because the organs affected by temperature were different in the two studies. In this study, SRP increased both the CE (**Figure 3F**) and *A*_max_ (**Figure 3D**) of lettuce plants, suggesting that the photosynthesis improvement correlated with Rubisco activity and RuBP regeneration, and thus improving the photosynthetic rate, which is in accordance with our previous results that CO_2_ assimilation efficiency of cucumber plants is promoted by Spd via increasing Rubisco activity and RuBP regeneration (Shu et al., 2012, 2014). These results suggested the existence of different mechanisms of *P*_N_ improvement in lettuce plants between RZC and SRP, the former was independent of Rubisco activity, while the latter owing to the enhancement of Rubisco activity.

### Chlorophyll Fluorescence Analysis Indicated that Both RZC and SRP Could Promote the Level of the PSII Photochemical Efficiency of Lettuce Plant, Thus Leading to Increase of Photosynthetic Efficiency

The influence of many environmental factors on photosynthesis can be evaluated by chlorophyll fluorescence parameters. In this study, F_v_/F_m_ (**Figure 5A**), F_v′_/F_m′_ (**Figure 5B**), and *q*N (**Figure 5F**) were not affected by RZC, similar to that there was no difference in F_v_/F_m_ measured from leaves of lettuce plants grown at both 20°C and hot ambient temperature from 26 to 35 and 28 to 41°C in January and June, respectively (He and Lee, 1998), suggesting that the enhancement of photosynthetic efficiency of lettuce plants was independent of light-harvesting and excessive energy dissipation. It also showed that the integrity of the photosynthetic apparatus was not damaged, which is consistent with the unchanged chlorophyll content (**Table 2**) because the plant chlorophyll content directly relates to photosynthetic apparatus integrity and light-harvesting (Tanaka and Tanaka, 2006). However, RZC increased PhiPSII (**Figure 5C**), PhiCO_2_ (**Figure 5D**), and *q*P (**Figure 5E**), suggesting the quantum yield of PSII electron transport, the quantum yield of carboxylation rate, and photochemical quenching in lettuce leaves improved when plants were exposed to a high-temperature season, which according to Zhang et al. (2008), who suggested that cooling root temperatures induced slight changes in PhiPSII and *q*P of six *Cucurbitaceae* species. The dynamic changes of chlorophyll fluorescence kinetic curves (**Figures 6A–C**) and response curves of fluorescence–CO_2_ (**Figures 6D–F**) revealed that *q*P, PhiPSII, and PhiCO_2_ were also improved by RZC. Calatayud et al. (2008) found that the PhiPSII and *q*P were higher in rose (*Rosa* × *hybrida* cv. Grand Gala) plants grown at cold solution of 10°C than that of 20°C, which meant that the majority of photons absorbed by PSII and used in photochemistry were promoted to increase the level of the photochemical efficiency of PSII. Therefore, RZC could promote quantum yields of both PSII electron transport and carboxylation rates of lettuce plant, leading to increase of photosynthetic efficiency. In this study, SRP did not affect F_v_/F_m_ (**Figure 5A**), F_v′_/F_m′_ (**Figure 5B**), and *q*N (**Figure 5F**), but increased PhiPSII (**Figures 5C** and **6B**), PhiCO_2_ (**Figures 5D** and **6C**), and *q*P (**Figures 5E** and **6A**), also suggesting that promotion of photosynthesis by SRP was dependent on the level of increase of the photochemical efficiency of PSII, but was independent on light-harvesting and excessive energy dissipation, this is in accordance with the observed photosynthesis improvement by the application of exogenous Put on cucumber plants under salt stress (Zhang et al., 2009; Shu et al., 2011).

### Improvement of *P*_N_ by Combination of RZC and SRP Correlated to Both CO_2_ Supply and CO_2_ Assimilation Enhancement

In this study, compared to control (30°C), combination of RZC and SRP improved *P*_N_ and *G*_s_ of lettuce plants, but not affected *C*i (**Figure 2**), suggesting improvement of *P*_N_ correlated to not only CO_2_ supply being enhanced by stomatal conductance but also CO_2_ assimilation enhancement, according to the determination of stomatal and non-stomatal limitation described by Farquhar and Sharkey (1982). Furthermore, apparent photosynthetic parameters such as *P*_Nmax_ (**Figure 3C**), *A*_max_ (**Figure 3D**), AQY (**Figure 3E**), and CE (**Figure 3F**), and chlorophyll fluorescences such as PhiPSII (**Figures 5C** and **6B**), PhiCO_2_ (**Figures 5D** and **6C**), and *q*P (**Figures 5E** and **6A**) were all enhanced by combination of RZC and SRP, compared to control and other treatments, suggesting that Rubisco activity, RuBP regeneration and the level of the photochemical efficiency of PSII were promoted, thus leading to increase of photosynthetic efficiency. As a result, plants growth was also promoted the most under combination of RZC and SRP treatment (**Table 1**) when the lettuce plants exposed to high temperature season.

## Conclusion

Both RZC and SRP effectively increased hydroponic lettuce plant growth and photosynthetic efficiency, but the mechanisms of photosynthesis improvement were different between the two treatments. The former improved photosynthesis of lettuce plants through increasing stoma conductance to enhance CO_2_ supply, thereby promoting photosynthetic electron transport activity and phosphorylation, which improved the level of the photochemical efficiency of PSII, rather than enhancing CO_2_ assimilation efficiency. The latter improved photosynthesis by enhancing CO_2_ assimilation efficiency, rather than stomatal regulation. Combination of RZC and SRP significantly improved *P*_N_ of lettuce plants in a high-temperature season not only due to improvement of CO_2_ supply but also CO_2_ assimilation enhancement. The enhancement of photosynthetic efficiency in both treatments was independent of altering light-harvesting or excessive energy dissipation.

## Author Contributions

We thank the numerous individuals who participated in this research. JS and SG conceived and designed the study. JS, NL, and HX conducted the experiments. TM contributed new reagents and analytical tools. JS and HX analyzed the data. JS and NL wrote the manuscript. All authors read and approved the manuscript.

## Conflict of Interest Statement

The authors declare that the research was conducted in the absence of any commercial or financial relationships that could be construed as a potential conflict of interest.
